# Whole-genome SNP analysis elucidates the genetic structure of Russian cattle and its relationship with Eurasian taurine breeds

**DOI:** 10.1186/s12711-018-0408-8

**Published:** 2018-07-11

**Authors:** Alexander A. Sermyagin, Arsen V. Dotsev, Elena A. Gladyr, Alexey A. Traspov, Tatiana E. Deniskova, Olga V. Kostyunina, Henry Reyer, Klaus Wimmers, Mario Barbato, Ivan A. Paronyan, Kirill V. Plemyashov, Johann Sölkner, Ruslan G. Popov, Gottfried Brem, Natalia A. Zinovieva

**Affiliations:** 1 L.K. Ernst Federal Science Center for Animal Husbandry, Dubrovitzy 60, Podolsk, Moscow Russia 142132; 2 Institute of Genome Biology, Leibniz Institute for Farm Animal Biology (FBN), 18196 Dummerstorf, Mecklenburg-Vorpommern Germany; 3 Department of Animal Sciences, Food and Nutrition, Università Cattolica del Sacro Cuore, via Emilia Parmense 84, Piacenza, Italy; 4 Russian Research Institute of Farm Animal Genetics and Breeding, Moskovskoe shosse 55a, St. Petersburg–Pushkin, Russia 196601; 5 Division of Livestock Sciences, University of Natural Resources and Life Sciences, Gregor-Mendel-Straße 33, 1180 Vienna, Austria; 6 Yakut Scientific Research Institute of Agriculture, 23/1, ul. Bestuzheva-Marlynskogo, Yakutsk, Sakha Republic Russia 677001; 7 Institute of Animal Breeding and Genetics, University of Veterinary Medicine, Veterinärplatz 1, 1210 Vienna, Austria

## Abstract

**Background:**

The origin of native and locally developed Russian cattle breeds is linked to the historical, social, cultural, and climatic features of the diverse geographical regions of Russia. In the present study, we investigated the population structure of nine Russian cattle breeds and their relations to the cattle breeds from around the world to elucidate their origin. Genotyping of single nucleotide polymorphisms (SNPs) in Bestuzhev (n = 26), Russian Black-and-White (n = 21), Kalmyk (n = 14), Kholmogor (n = 25), Kostromsky (n = 20), Red Gorbatov (n = 23), Suksun (n = 20), Yakut (n = 25), and Yaroslavl cattle breeds (n = 21) was done using the Bovine SNP50 BeadChip. SNP profiles from an additional 70 breeds were included in the analysis as references.

**Results:**

The observed heterozygosity levels were quite similar in eight of the nine studied breeds (H_O_ = 0.337–0.363) except for Yakut (Ho = 0.279). The inbreeding coefficients *F*_IS_ ranged from -0.028 for Kalmyk to 0.036 for Russian Black-and-White and were comparable to those of the European breeds. The nine studied Russian breeds exhibited taurine ancestry along the C1 axis of the multidimensional scaling (MDS)-plot, but Yakut was clearly separated from the European taurine breeds on the C2 axis. Neighbor-Net and admixture analyses, discriminated three groups among the studied Russian breeds. Yakut and Kalmyk were assigned to a separate group because of their Turano-Mongolian origin. Russian Black-and-White, Kostromsky and Suksun showed transboundary European ancestry, which originated from the Holstein, Brown Swiss, and Danish Red breeds, respectively. The lowest level of introgression of transboundary breeds was recorded for the Kholmogor, Yaroslavl, Red Gorbatov and Bestuzhev breeds, which can be considered as an authentic genetic resource.

**Conclusions:**

Whole-genome SNP analysis revealed that Russian native and locally developed breeds have conserved authentic genetic patterns in spite of the considerable influence of Eurasian taurine cattle. In this paper, we provide fundamental genomic information that will contribute to the development of more accurate breed conservation programs and genetic improvement strategies.

**Electronic supplementary material:**

The online version of this article (10.1186/s12711-018-0408-8) contains supplementary material, which is available to authorized users.

## Background

Livestock breeding is an important sector of agriculture in Russia and is inextricably linked with the historical, social, cultural, and climatic features of the regions of this country. The beginning of cattle breeding and spreading throughout Russia is associated with the relocation of ancient Slavic tribes during the sixth century [1]. The history of Russia has led to the formation of cattle populations that are well adapted to the local climatic environment and economic conditions of certain regions [2]. In the second decade of the twentieth century, based on the territorial principle and phenotypic traits, several large groups of cattle populations were formed in the USSR—the so-called Russian tribes (currently defined as breeds)—”which acquired considerable importance, and were of great economic interest and became the subject of… breeding” [2]. Large-scale breeding of cattle in Russia began in the 1920–1940s, when the first herd books were published and the first breeds were officially recognized, including Russian Black-and-White (in 1925), Yaroslavl (1925), and Kholmogor (in 1927), which originated from the Northern Great Russian land cattle; the Red Gorbatov (in 1926), Bestuzhev (in 1928), and Suksun (in 1943) breeds of red cattle; Yakut (in 1929), which was native to Siberia; Kalmyk (in 1934), which was bred by the nomadic people of the southern steppe of Russia; and the Kostromsky breed of brown cattle (in 1943) (for a short description of breeds [see Additional file 1: Table S1]). Due to the unorganized importation of almost all cattle breeds that were bred in Western Europe from the first quarter of the eighteenth century to the early twentieth century [3, 4], the genetic origin of the Russian cattle breeds is not entirely clear. There are different viewpoints concerning the contribution of imported breeds in the formation of the Russian cattle population. While some authors claim a composite origin of most of the Russian cattle breeds [5, 6], others insist on only minor contributions from the foreign breeds in the development of the Russian cattle population [7, 8].

Many studies have been undertaken to clarify the demographic history of cattle breeds in the European, Asian, and North and South American countries, as well as in Africa [9–14], but little is known about the genetic origin of cattle breeds in Russia. Different types of genetic markers have been applied to reconstruct the demographic history of cattle breeds worldwide. Microsatellites were used to verify the hybrid origin of the Near-Eastern cattle breeds [15, 16], elucidate the different histories of the Mediterranean and Northern European cattle populations, [17] and clarify the classification of the Eurasian cattle breeds [18]. Mitochondrial DNA polymorphisms and microsatellites were successfully applied to study the genetic diversity and the genetic structure of several Russian cattle breeds [18–23]. However, the genetic relationships between some breeds remain ambiguous owing to the lack of discriminatory power of these genetic markers [24]. The full sequence of the bovine genome was completed in 2009 [25, 26], which led to the identification of several hundred thousands single nucleotide polymorphisms (SNPs) that have been successfully used in livestock studies at the genome-wide level [10, 27–31]. Whole-genome SNP analyses were successfully applied to characterize the diversity and population structure of the Russian cattle breeds, but these studies were either limited to a few breeds [32] or did not compare the Russian cattle breeds with the worldwide breeds [33].

Here, a genome-wide comparative study of nine native and locally developed Russian cattle breeds was performed with the aim to characterize their genetic architecture and clarify the contribution of the worldwide breeds to their origin.

## Methods

### Sample description and DNA extraction

**Fig. 1:**
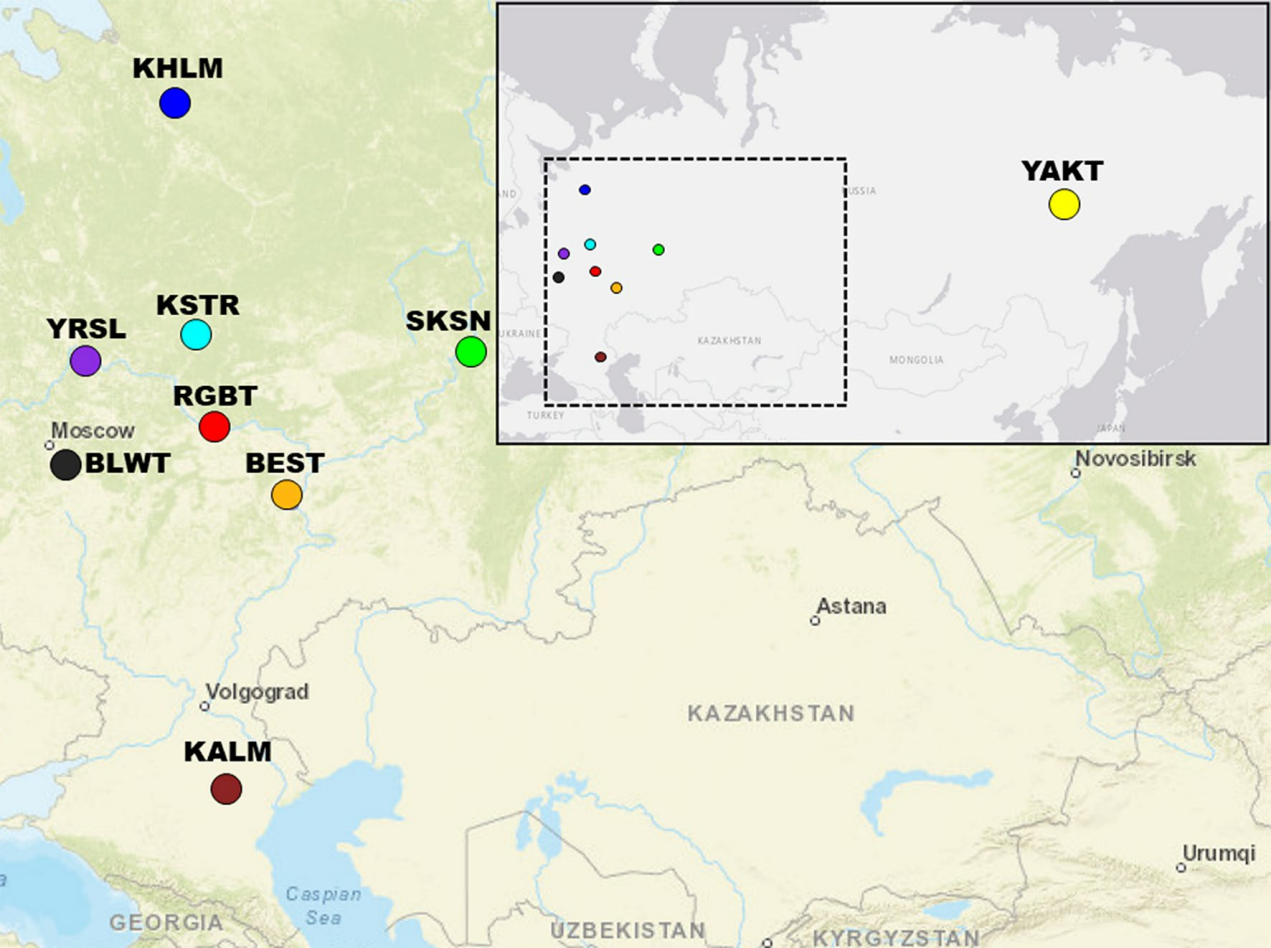
Map [34, 35] illustrating the geographical origin of the nine Russian cattle breeds included in this study. Bestuzhev—BEST (orange), Black-and-White—BLWT (black), Kalmyk—KALM (brown), Kholmogor—KHLM (blue), Kostromsky—KSTR (light blue), Red Gorbatov—RGBT (red), Suksun—SKSN (green), Yaroslavl breeds—YRSL (purple), and Yakut—YAKT (light brown)

A total of 195 samples (sperm or blood) collected from nine locally derived Russian cattle breeds, namely, Bestuzhev (BEST, n = 26), Russian Black-and-White (BLWT, n = 21), Kalmyk (KALM, n = 14), Kholmogor (KHLM, n = 25), Kostromsky (KSTR, n = 20), Red Gorbatov (RGBT, n = 23), Suksun (SKSN, n = 20), Yakut (YAKT, n = 25), and Yaroslavl (YRSL, n = 21), were analysed. Blood samples from eight of the nine breeds (excluding Russian Black-and-White) were collected from purebred herds during the 2004–2016 period, whereas Russian Black-and-White was represented by semen samples (kept in a genetic resources’ collection; unique collection number 663.00.X3057) of bulls born in the 1970–1980s. Blood samples were collected during routine veterinary procedures. Sperm samples were provided by the artificial insemination (AI) stations according to specific scientific collaboration agreements. Figure 1 shows a map [34, 35] that indicates the regions of sample collection.

Genomic DNA was extracted using Nexttec columns (Nexttec Biotechnology GmbH, Germany) and following the instructions of the manufacturer. DNA concentration was estimated by measuring the absorbance at 260 nm, and DNA quality was determined by separation on agarose gels. Whole-genome SNP genotyping was performed using the Bovine SNP50 BeadChip (Illumina, San Diego, CA, USA).

The genotypes of 746 individuals representing 70 breeds distributed around the world were included in the dataset (see Additional file 2: Table S2). Genotyping data of breeds from the former USSR were from the SNP library of the L.K. Ernst Federal Science Center of Animal Husbandry, whereas those of the other worldwide breeds were obtained from publicly available sources [36].

### Selection of SNPs for analysis

To assess the accuracy and efficiency of SNP genotyping, the GenCall (GC) and GenTrain (GT) scores were used. A cut-off of 0.5 for both the GC and GT scores was applied to determine the valid genotypes for each SNP [37]. SNP quality filtering was performed using PLINK v 1.07 [38]. SNPs for which < 90% of individuals were genotyped (–geno 0.1), that had a minor allele frequency (MAF) lower than 5% (–maf 0.05) and that departed from the Hardy–Weinberg equilibrium with a *p* value < 10^−6^ (–hwe 1e-6) were excluded from further analyses. LD filtering was performed by removing SNPs with the squared correlation coefficient between the two alleles of the two SNPs (*r*^2^) higher than 0.5 within 50-SNP sliding windows with each window overlapping by 5 SNPs (–indep-pairwise 50 5 0.5). SNPs located on sex chromosomes or with unknown map positions were also removed. Individuals for which < 90% of SNPs were genotyped (–mind 0.1) were removed. A Hardy–Weinberg equilibrium test was not performed for comparisons with the worldwide breeds because too many SNPs would have been excluded due to the Wahlund effect [39].

### Estimation of genetic diversity within breeds

Within-breed genetic variability was evaluated using the R package ‘diveRsity’ [40] to calculate the rarefied allelic richness (A_R_), observed (H_O_) and unbiased estimate of the expected heterozygosity (H_E_) [41], and inbreeding coefficient (*F*_IS_).

### Genetic differentiation of breeds and population structure analysis

To characterize the genetic differences between the Russian breeds, we calculated the overall *F*_ST_ value [42] using the R package ‘diveRsity’ [40]. Breed differentiation was evaluated using pairwise *F*_ST_ values [42] and multidimensional scaling (MDS). Pairwise *F*_ST_ values were also calculated with the R package ‘diveRsity’ [40]. MDS analysis based on pairwise identical-by-state (IBS) distances was performed with PLINK 1.07 (–cluster, –mds-plot 4) and visualized with the R package “ggplot2” [43].

To reduce the bias that is caused by longest genetic distances (*F*_ST_) and inbreeding [44], we excluded the American, African, Australian as well as the Chinese and Indonesian breeds from the dataset and selected only the Eurasian breeds for the network and admixture analysis. In total, the SNP profiles of 45 breeds from Eurasia including nine Russian breeds were pooled. A pairwise matrix of *F*_ST_ values [42] was used to construct networks of breed relationships [45], using the program SplitsTree version 4.14.5 [46]. Genetic admixture analysis was carried out using Admixture 1.3 [47]. Data was visualized using the R package “pophelper” [48]. We evaluated the K values (the number of assumed ancestral populations), ranging from 1 to 40, along with their respective cross-validation (CV) errors.

### Estimation of the effective population size

Trends in historical effective population size (Ne) were estimated from linkage disequilibrium (LD) as implemented in *SNeP* v1.11 [49]. Default parameters were applied, except for the correction for sample size, the occurrence of mutation (α = 2.2; [50]), and the recombination rate modifier, according to Sved and Feldman [51]. Following Kukučková et al. [52], the current effective population size (Ne_0_) was inferred based on the results of linear regression analysis performed on Ne estimates ranging from 10 to 60 generations ago. A “Ne changing ratio” (NeC) analysis was used as a proxy of the speed in Ne changes in the 13 most recent generations. The slope of each segment that links a pair of neighbouring Ne estimates was calculated and normalized using the median of the 13 most recent Ne estimates.

R version 3.3.2 was used as an instrument for creating input files [53].

## Results

### SNPs characteristics and within-breed genetic diversity

After quality control and filtering, 35,874 SNPs remained for further analyses. The summary statistics for genetic diversity are in Table 1. The A_R_ and H_O_ values were similar in eight of the nine breeds analysed (A_R_ ranged from 1.918 to 1.958 and H_O_ from 0.337 to 0.366), except for the Yakut breed, for which a significantly lower level of variability was observed (A_R_ = 1.780 and Ho = 0.279). Six of the nine breeds analysed had a significant (95% CI) excess of heterozygotes, while in the three remaining breeds (Bestuzhev, Russian Black-and-White, and Yaroslavl) a deficiency in heterozygotes was identified. The largest excess in heterozygote was detected in Kholmogor (F_IS_ = − 0.028), while the Russian Black-and-White breed was characterized by the largest deficiency in heterozygotes (*F*_IS_ = 0.036) (Table 1). The current effective population size estimated from LD ranged from Ne_0_ = 63 (Red Gorbatov) to Ne_0_ = 161 (Bestuzhev).

**Table 1:** Summary statistics for the genetic diversity of nine Russian cattle breeds

Breed abbreviation	n	A_R_	H_O_ (± 0.001)	H_E_ (± 0.001)	Ne_0_ (β_1_)	*F*_IS_ [95% CI]
BEST	26	1.956 ± 0.001	0.357	0.359	161 ± 5 (10)	0.004 [0.002; 0.006]
BLWT	21	1.949 ± 0.001	0.341	0.355	115 ± 6 (10)	0.036 [0.034; 0.039]
KALM	14	1.959 ± 0.001	0.363	0.360	115 ± 13 (21)	− 0.006 [− 0.009; − 0.003]
KHLM	25	1.923 ± 0.001	0.350	0.340	65 ± 2 (6)	− 0.028 [− 0.030; − 0.026]
KSTR	20	1.918 ± 0.001	0.342	0.337	88 ± 2 (7)	− 0.013 [− 0.015; − 0.011]
RGBT	23	1.925 ± 0.001	0.338	0.336	63 ± 2 (5)	− 0.006 [− 0.008; − 0.003]
SKSN	20	1.953 ± 0.001	0.366	0.360	75 ± 3 (9)	− 0.018 [− 0.018; − 0.014]
YAKT	25	1.780 ± 0.002	0.279	0.278	64 ± 2 (3)	− 0.003 [− 0.005; − 0.001]
YRSL	21	1.927 ± 0.001	0.337	0.340	99 ± 3 (5)	0.007 [0.007; 0.009]

A_R_, rarified allelic richness; H_O_, observed heterozygosity; H_E_, unbiased expected heterozygosity; Ne_0_, current effective population size inferred as the intercept and related standard error as well as the slope of the regression (β_1_); *F*_IS_, inbreeding coefficient; CI, confidence interval (threshold values are showed in square brackets); for the full definitions of breeds (see Additional file 1: Table S1)

### Genetic differentiation and relationships between breeds

**Fig. 2:**
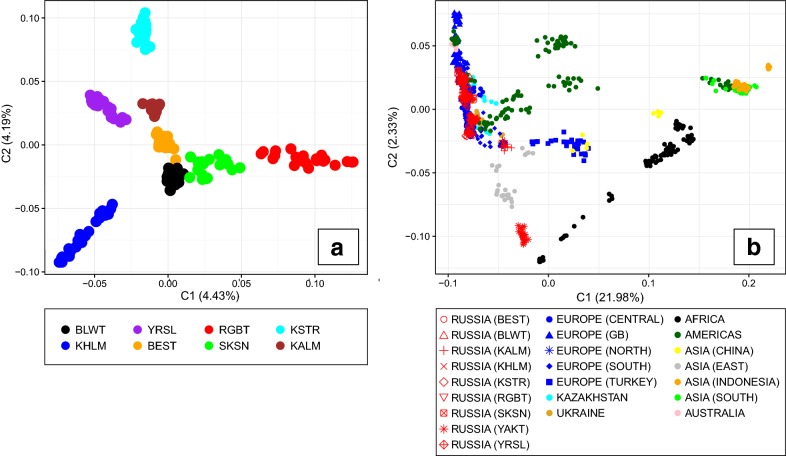
Multi-dimensional scaling (MDS) analysis of native and locally developed Russian cattle breeds. **a** Analysis of the breeds from the European part of Russia. The colours are identical to the colours of the breeds’ regions of origin in Fig. 1. **b** Analysis of the worldwide cattle breeds. The Russian breeds are denoted in red colour. For the full definition of breeds (see Additional file 1: Table S1 and Additional file 2: Table S2)

Multidimensional scaling revealed the clear differentiation of breeds from the European part of Russia (Fig. 2a). Component 1 (C1) accounted for 4.43% of the variability and discriminated the red breeds (Red Gorbatov and Suksun) from those derived from the Northern Great Russian land cattle (Yaroslavl + Kholmogor). Component 2 (C2) accounted for 4.19% of the variance and discriminated the breeds that are less related to Dutch cattle (Kholmogor, Yaroslavl + Kalmyk, and Kostromsky) from those showing higher levels of Dutch cattle introgression followed by Holstein cattle introgression. At the global level (Fig. 2b), breeds were separated on the X-axis into two main groups according to the origin of their species: taurine *(Bos taurus)* and indicine cattle *(Bos indicus)*. Most of the studied Russian breeds (Russian Black-and-White, Bestuzhev, Kholmogor, Red Gorbatov, Suksun, and Yaroslavl) formed a cluster that overlapped with the breeds from Northern Europe and Great Britain as well as those from Southern Europe (Kostromsky and Kalmyk). The Yakut breed exhibited taurine ancestry along the C1 axis, but was clearly separated from the European taurine breeds on the C2 axis.

**Fig. 3:**
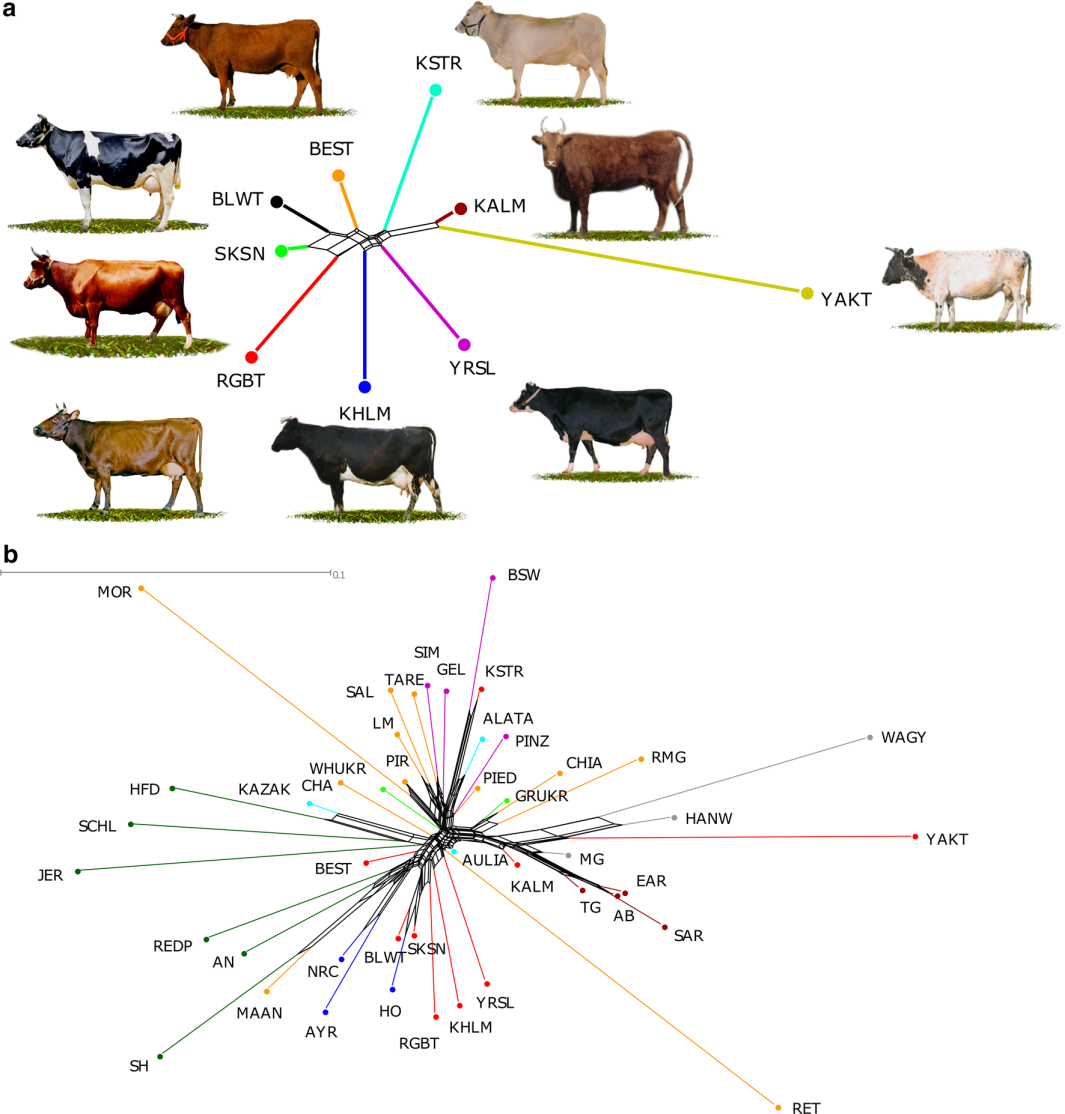
Neighbor-Net dendrogram constructed from pairwise matrix of *F*_ST_ values [42]. **a** Analysis was carried out for nine Russian cattle breeds. The colours are identical to the colours of the breeds’ regions of origin in Fig. 1. **b** Analysis of the Eurasian cattle breeds. The different colours indicate the geographical origin of breeds: Russia—red, former USSR countries Kazakhstan and Ukraine—light blue and light green, respectively, Great Britain—green, Northern Europe—blue, Central Europe—purple, Southern Europe—orange, Turkey—dark red, Western Asia—grey. For the full definition of breeds (see Additional file 1: Table S1 and Additional file 2: Table S2)

The Neighbor-Net (Fig. 3a) analysis confirmed the clear differentiation of all the studied Russian cattle breeds. The most distant cluster included the Yakut and Kalmyk breeds, which belong to the Turano-Mongolian root breeds (*Bos taurus turano*-*mongolicus*). Among the remaining breeds, the Red cattle breeds clustered together at the opposite end, and among these the Red Gorbatov breed was the most divergent. The Russian Black-and-White breed was integrated in the Red breeds’ cluster between the Suksun and Bestuzhev breeds, which indicates the presence of possible common ancestry. The intermediate positions between the Turano-Mongolian breeds and the Red cattle breeds were occupied by branches comprising the native Yaroslavl and Kholmogor breeds, and the Kostromsky breed on the opposite end. Neighbor-Net of Eurasian breeds (Fig. 3b) showed a three-cluster structure: (1) a first cluster joined the breeds from Great Britain and Northern Europe and included six Russian breeds (Bestuzhev, Russian Black-and-White, Kholmogor, Red Gorbatov, Suksun, and Yaroslavl); (2) a second cluster was comprised of Central European and Southern European breeds including Kostromsky, which formed the common branch with Brown Swiss cattle; and (3) Turano-Mongolian root breeds (including Kalmyk and Yakut), and Podolian cattle breeds branched as a third cluster.

### Population structure of the Russian cattle breeds

**Fig. 4:**
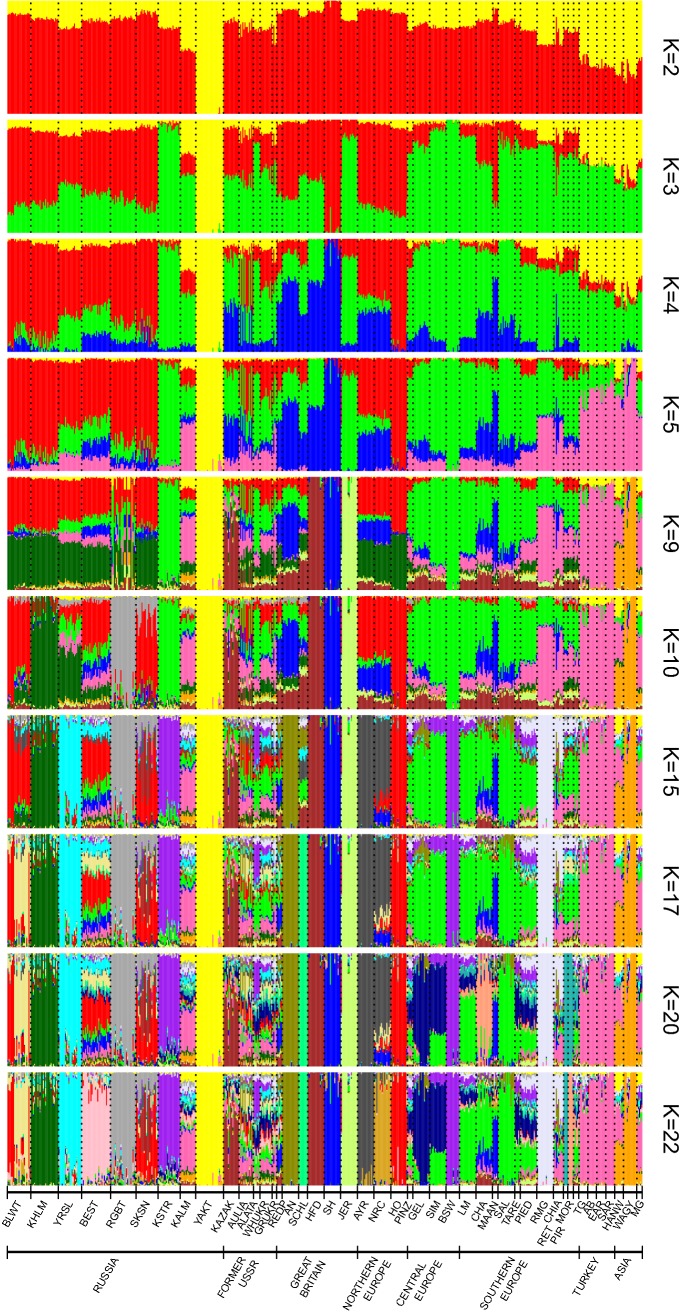
Bar plot showing the extent of admixture of the Russian breeds with 36 Eurasian breeds. Breeds are grouped according to their origin: Russia, former USSR countries, Great Britain, Northern Europe, Central Europe, Southern Europe, Turkey, and Eastern Asia. For the full definitions of breeds (see Additional file 1: Table S1 and Additional file 2: Table S2)

Population structure of the Russian cattle breeds and the possible introgression of other Eurasian breeds were inferred by admixture analysis (Fig. 4). We used 10 clustering solutions at K = 2–5, 9, 10, 15, 17, 20 (the lowest cross-validation value (Cv) = 0.587; [see Additional file 3: Figure S1]), and 22, which showed the Russian ancestry of the studied breeds (at lower K values) and that they shared ancestry with other Eurasian breeds. At K = 2, Yakut clustered separately from the other Eurasian breeds, while the majority of the other Russian breeds (except Kalmyk) showed a relatively low Yakut genomic component. Among the studied Russian breeds, the highest Yakut genomic component was found in Kalmyk, which persisted at higher K values. At K = 3, Kostromsky clustered separately from the other Russian breeds with a clustering component mostly shared with breeds of Central European origin. The structure of the cluster of six breeds (Bestuzhev, Kholmogor, Red Gorbatov, Suksun, Yaroslavl, and Yakut) was similar to that of the Northern European breeds with a slightly higher percentage of Yakut genomic component. At K = 4, the Holstein breed formed its own cluster, with all six above-mentioned Russian breeds sharing a high percentage of Holstein ancestry. Only four of these breeds (Bestuzhev, Kholmogor, Red Gorbatov, and Yaroslavl) retained Yakut specific components. At K = 5, all studied breeds from the European part of Russia revealed clustering components that were consistent with Turkish—West Asian roots. At K = 9, Red Gorbatov showed an extremely heterogeneous genetic structure, with all of the nine putative ancestry sources hardly distinguishable. At K = 10, Red Gorbatov and Kholmogor clustered separately, while Russian Black-and-White, Bestuzhev, and Suksun maintained the high percentage of Holstein genetic roots. At K = 15, 17 and 22, Yaroslavl, Russian Black-and-White (partly), and Bestuzhev, respectively, were assigned to their own clusters, whereas, Kalmyk retained a heterogeneous genetic structure.

### Effective population sizes

**Fig. 5:**
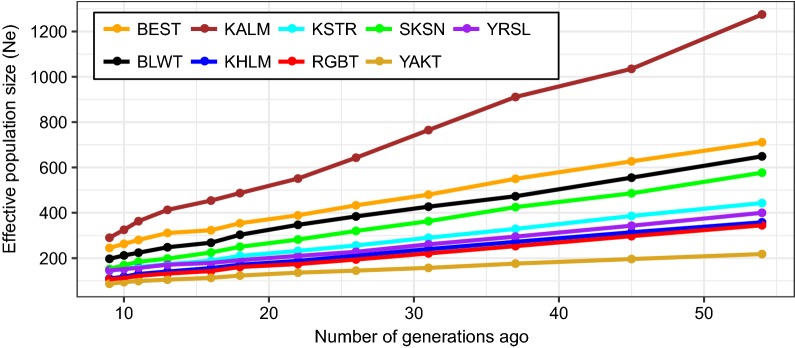
Historical effective population size (Ne) from approximately 50 generations ago based on linkage disequilibrium (LD) estimates. For the full definition of breeds (see Additional file 1: Table S1)

Most of the breeds showed a slow decline in the effective population size over time (Fig. 5). The Ne values preceding such time points ranged from 100 to 700 for most of the breeds, except for Kalmyk, which was around 1200 at 50 generations ago. The Ne_0_ values ranged from 63 to 161, with Red Gorbatov and Bestuzhev showing the lowest and highest values, respectively. The slope value (β1) of most of the linear regression models ranged from 5 to 10, whereas Kalmyk and Yakut had values of 21 and 3, respectively (Table 1) and (see Additional file 4: Figure S2). All linear regression models had a high coefficient of determination (*r*^*2*^ > 0.99).

## Discussion

Understanding the genetic diversity and population structure of local cattle breeds is necessary for their genetic improvement and the development of effective conservation programs as well as for understanding their capacity to survive under certain environmental conditions [54, 55]. Numerous studies have been undertaken to characterize the Russian cattle breeds using mitochondrial DNA polymorphisms [19], single protein-coding genes [56] and multiple microsatellite loci [11, 18–21, 57]. To elucidate the origin of the Russian cattle population, we investigated the genetic diversity and population structure of nine Russian cattle breeds, including eight breeds from the European part of Russia and one breed native to Siberia (Yakut), and their relationship with the cattle breeds from around the world at the whole-genome level using a set of 35,874 polymorphic SNPs from the Bovine SNP50 K BeadChip (Illumina, Inc., San-Diego, USA). An average *F*_ST_ value of 0.0982 was obtained for the Russian breeds, which suggests that 9.8% of the variability was due to between-breed differentiation and the remaining 90.2% was due to allelic variations within breeds. The level of genetic variability (H_E_ = 0.355–0.360; A_R_ = 1.949–1.959) was highest in the Bestuzhev, Russian Black-and-White, Kalmyk, and Suksun breeds, which indicates the participation of several genetically distinct breeds in their development. The level of genetic variability observed in eight Russian breeds, excluding Yakut, was similar to that in other taurine breeds [32, 58]. The lowest level of genetic diversity (H_E_ = 0.278; A_R_ = 1.780; Ne_0_ = 64) was recorded for the Yakut breed of Siberia, which could be due to the extremely small size of the population (600 individuals in 2015, [see Additional file 1: Table S1]). Among the focal Russian breeds, the *F*_IS_ values ranged from − 0.028 (Kalmyk) to 0.036 (Black-and-White). Our observations were in agreement with Gautier et al. [59], who reported similar *F*_IS_ values for 28 of the 29 European breeds in their study (*F*_IS_ ranging from − 0.040 to 0.027) based on 44,706 SNPs. Five of the studied Russian breeds had *F*_IS_ values close to zero (ranging from − 0.006 to 0.007), while four breeds showed relatively higher deviations. The Kholmogor, Kostromsky and Suksun breeds were characterized by an excess in heterozygotes (*F*_IS_ ranging from − 0.013 to − 0.028). For the Kostromsky and Suksun breeds, the negative *F*_IS_ values were probably due to recent crossbreeding with Brown Swiss (for Kostromsky), and Danish Red and Red Holstein cattle (for Suksun). For the Kholmogor breed, the excess in heterozygotes may be associated with breeding schemes that use bulls of various genetic origins. We observed a deficiency in heterozygotes (*F*_IS_ = 0.036) in Russian Black-and-White cattle, which was probably due to the greater use of a limited number of bulls and higher selection pressures for a limited number of productive traits compared to other breeds.

### On the origin of the Russian cattle breeds

By applying a variety of statistical estimations to whole-genome SNP data, we unravelled the genetic structure within Russian cattle breeds. Furthermore, we assessed the distinctiveness of Russian cattle in a scenario including world-wide cattle. The results of the admixture, MDS-plot, and Neighbour-Net analyses were consistent regarding the genetic relationship and population structure patterns in the Russian breeds analysed in this study (Figs. 2a, 3, 4).

We identified three major groups within the studied Russian breeds, based on their historical origin and degree of conservation of ancestral genomic components. In contrast, based on results of a principal component analysis, Yurchenko et al. [60] grouped the Russian cattle breeds into four clusters, by combining the breeds that are strongly affected by transboundary breeds with those that have maintained a considerable degree of the Russian specific component. Such a clustering reflects mainly the earlier demographic history of Russian cattle breeds and less the recent migration events.

In our study, we included in the first group the Yakut and Kalmyk breeds, which clustered with breeds of Turano-Mongolian origin (Fig. 3b). Yakut was the most distinct breed among those analysed here, which is in agreement with the results of Yurchenko et al. [60]. Yakut is characterized by a high degree of differentiation from the other Russian breeds (*F*_ST_ ranging from 12.9 to 19.0%) and from other Eurasian breeds (*F*_ST_ ranging from 13.6 to 27.5%) (see Additional file 5: Table S3). Among all the breeds studied here, Yakut appeared to be closer to the Russian Kalmyk, Mongolian, and Korean Hanwoo breeds, although the differences in pairwise *F*_ST_ between them were relatively large (*F*_ST_ ranging from 12.9 to 14.2%) (see Additional file 5: Table S3). The Kalmyk breed displayed a very complex genetic structure, although it maintained the highest percentage of Yakut genomic component among the Russian breeds (Fig. 4). This breed originated from cattle that were bred by nomadic people who inhabited the southern steppe regions of Russia. The Kalmyk breed appeared to be genetically close to the Mongolian (*F*_ST_ = 3.1%) and Gray Ukrainian breeds (*F*_ST_ = 3.3%) (see Additional file 5: Table S3), which suggests a common ancestor. In the MDS plot, this breed was located between the breeds from south Europe and East Asia (Fig. 2b), which indicates that they contributed to the development of the Kalmyk breed. This breed showed gene flow signals that originated from several other Russian, Southern European, and West Asian breeds (Fig. 4), in agreement with findings in previous studies [29, 31, 60–62] and historical records on the breed [63, 64]. Our results suggest that the Yakut and Kalmyk breeds constitute a unique Turano-Mongolian genetic resource in the Russian cattle breed pool, and therefore deserve attention for their conservation; however, gene flow from European breeds to the Kalmyk breed cannot be completely excluded and further analyses are necessary for a more precise assessment.

The second group clustered together the Black-and-White, Kostromsky, and Suksun breeds, which result from multiple crossbreeding with transboundary European breeds. *F*_ST_, Neighbor-Net and admixture results indicate a high percentage of Holstein ancestry in the Russian Black-and-White breed (see Additional file 5: Table S3) and (Figs. 3b, 4). Traces of other ancestors were revealed in a separate clustering of most of the Black-and-White animals at K = 17 (Fig. 4). For the Kostromsky breed, we observed a high degree of identity with Brown Swiss cattle, as suggested by the low *F*_ST_ value (5.2%) in the pairwise comparison (see Additional file 5: Table S3), the neighboring locations at the Neighbor-Net (Fig. 3b), and the high degree of Brown Swiss specific component in the model-based clustering (Fig. 4). This observation is in line with the history of the breed, which was developed by multiple backcrossing with Brown Swiss bulls [4, 65]. The Suksun breed displayed admixture signals of Northern European cattle, which probably reflect the active use of Danish Red cattle in the earliest stages of its development [65]. This was further supported by microsatellite-based studies of Eurasian cattle breeds, in which the Suksun breed clustered together with Danish Red cattle [18, 61]. Thus, our data confirm that, in the past, expansion events of the Northern-European breeds such as Danish Red, Holstein–Friesian, and Ayrshire occurred in Russia, as previously highlighted by large-scale microsatellite studies [18, 61].

The third cluster included the Kholmogor, Yaroslavl, Red Gorbatov, and Bestuzhev breeds, which were characterized by a reduced influence of non-Russian genetics components. Kholmogor is a breed of tall cattle that inhabit the islands in the upper reaches of the Northern Dvina River in Northern Russia. The first records of the presence of cattle in this area date back to the times of Ivan the Terrible (sixteenth century) [4]. It is known that Dutch cattle were provided to the inhabitants of the fertile meadows in Kholmogor in the 1760s under the order of Empress Catherine II [66]. Breeding of cattle in the Yaroslavl region (near Moscow, and the breed that was named after this region) was developed in the seventeenth and eighteenth centuries to supply the citizens of Moscow with dairy products [4]. Dutch, Tyrolean, Angeln, Simmental, and Kholmogor cattle were brought in small quantities to the Yaroslavl’s breeding zone at different times. However, after 1882–1883, cattle were no longer imported to this region of Russia [67]. The cline-like variation within the native Yaroslavl and Kholmogor breeds towards the Black-and-White breed (Fig. 2a) suggests a reduced contribution of Dutch cattle during the formation of the breeds, although they are separated by a significant genetic distance (see Additional file 5: Table S3). Our data show that a Russian genetic component is present in the Kholmogor and Yaroslavl breeds (*F*_ST_ values between these breeds and Holstein were 8.5 and 9.1%, respectively; [see Additional file 5: Table S3]), which clearly formed private branches on the Eurasian Neighbor-Net tree (Fig. 3b) and clustered separately in the admixture plot (Fig. 4). Our findings are consistent with those of several previous studies that suggested a small contribution of foreign breeds in the development of the Kholmogor and Yaroslavl breeds [60, 64, 68, 69], but contradict the findings of a microsatellite-based clustering analysis [18] that suggested the composite origin of these breeds but the authors themselves indicate the ambiguous nature of their results. Further studies are necessary to better decipher the history of these two aboriginal Russian cattle breeds.

We observed a high degree of divergence between Red Gorbatov (a breed of red cattle distributed on the banks of the Volga River) and the other breeds. The Red Gorbatov breed has its own branch within the British-Northern European cluster in the Eurasian Neighbor-Net tree (Fig. 3b) and clusters separately in the admixture-plot (Fig. 4). At the global level, Red Gorbatov seem to be closer to the Southern European breeds—Piedmontese (*F*_ST_ = 7.7%) and Pirenaica (*F*_ST_ = 8.1%) (see Additional file 5: Table S3), which reflects the contribution of Tyrolean cattle in the improvement of both breeds during the nineteenth century [64]. Most likely, this contribution came from the Tux-Zillertal breed, which was widespread in Tyrol because of its adaptability and good productive capacity in the presence of poor forage resources. At the agricultural exhibition of 1855 in Tyrol, Tux-Zillertal cattle were presented as a Tyrolean breed [70]. Interestingly, our findings contradict the previous observation by Felius [69], who classified the Red Gorbatov breed as derived from Alpine cattle breeds. We infer that during the century-long history of breed formation without cross-breeding, the Red Gorbatov breed developed its own genetic and phenotypic characteristics, which made it most suitable to the specific environmental conditions in Central Russia. We assigned Bestuzhev cattle to the third group due to its unique genetic identity (Figs. 3b, 4 at K = 22) in spite of its composite origin [65].

### Effective population size

Inference of the trends in Ne showed an overall decline of Ne for all the cattle breeds analyzed in this study. The inferred sizes of the current effective populations (Ne_0_) showed that several breeds approached a Ne_0_ of ~ 50 (Red Gorbatov, Yakut, Kalmyk; Table 1), which is generally considered to be a danger threshold in terms of species survivability to adaptation challenges [71]. Analysis of the ratio of Ne change revealed a peak at approximately 18 generations ago for almost all the breeds, which is most likely a reflection of the World War I and II crisis. The peak at 10 generations ago occurred in the 1960s, when artificial insemination began. For the Kalmyk breed, we observed a peak at approximately 45 to 50 generations ago (see Additional file 4: Figure S2), which could coincide with the period of the mid-eighteenth century when the forces of the Russian Empire turned traditional Kalmyk grazing lands into hayfields and passed them under the plough, which drove the Kalmyk people to a less fertile steppe and resulted in a drastic decline in the number of their livestock. Moreover, those events led to an exodus in 1771, during which the Kalmyk people lost most of their livestock [72]. The peak at approximately 30 generations ago (see Additional file 4: Figure S2) was probably due to the dramatic decline in the number of Kalmyk livestock during the 1830s, which was caused by the severe winter of 1833. According to official information, the number of cattle dropped from 124,690 in 1827 to 33,308 in 1837 [73].

## Conclusions

In this paper, we present the genetic characterization of nine native and locally developed Russian cattle breeds. Based on whole-genome SNP data analysis, we identified three distinct groups among the Russian breeds included here. The first cluster contained the Yakut and Kalmyk breeds of Turano-Mongolian origin, which were the most distant from the other breeds. The Russian Black-and-White, Kostromsky, and Suksun cattle clustered as a second group and represent breeds that were developed by multiple crossbreeding with West European transboundary breeds (Holstein, Brown Swiss and Danish Red, respectively). The third group comprised the Kholmogor, Yaroslavl, Red Gorbatov, and Bestuzhev breeds, which are those that have been the least influenced by introgression with non-Russian breeds, and thus they represent the authentic genetic resources. Such results provide a significant contribution to our understanding of the origin and modern genetic composition of the Russian cattle breeds, and provide the basis for developing more accurate programs for their conservation and genetic improvement.

## Additional files

**Additional file 1: Table S1.** Short description of the local Russian breeds under study. This table provides information about the Russian cattle breeds used in this study, including the year of the breeds’ official recognition, dynamics of their population size, the productivity type, and a short description of the breeds’ history.

**Additional file 2: Table S2.** Description of the reference samples included in the analyses. This table provides information about the reference samples used in this study including the sample size, country of origin, and the references where the genotypic data were previously published.

**Additional file 3: Figure S1.** CV-error according to the K-number. This graph shows the CV-error for the number of clusters (K) from 2 to 40.

**Additional file 4: Figure S2.** Trend in slope changes of historical effective population size (Ne). This graph shows the trend in slope changes of the historical effective population size (Ne) for the period starting approximately 70 generations ago. For the full definition of breeds, see Table S1 [see Additional file 1: Table S1].

**Additional file 5: Table S3.** Pairwise Wright fixation index (*F*_ST_). This table provides the values of Wright’s fixation index (*F*_ST_) at pairwise comparisons between the nine Russian cattle breeds and worldwide reference breeds. For the full definition of breeds, see Tables S1 and S2 [see Additional file 1: Table S1 and Additional file 2: Table S2].
