# Supplementary material for: Whole-genome SNP analysis elucidates the genetic structure of Russian cattle and its relationship with Eurasian taurine breeds
Source: Genet Sel Evol. 2018 Jul 11;50:37. doi: 10.1186/s12711-018-0408-8 (PMC6042431; doi:10.1186/s12711-018-0408-8)
Supplement: Supplementary file 1 — Additional file 1: Table S1. Short description of the local Russian breeds under study. This table provides information about the Russian cattle breeds used in this study, including the year of the breeds’ official recognition, dynamics of their population size, the productivity type, and a short description of the breeds’ history. [file 12711_2018_408_MOESM1_ESM.pdf]

Supplementary Materials

Table S1: Short description of the local Russian breeds under study.

Breed	Abbreviation	Status	The issue of the first herd book, year	Population size by year, in thousands heads			Productivity type	The region of origin, the breeds involved in breed development
				1960	1991	2015		
Bestuzhev	BEST	locally developed	1928	902.0	618.9	24.3	dairy	Central Russia, Ulyanovsk region; improvement of local cattle, which inhabited the banks of Volga river by Shorthorn, Dutch Holstein, Wilstermarsch and Simmental cattle
Black-and-White	BLWT	locally developed	1925	2167.0	8867.2	1649.6	dairy	Central part of Russia, the Volga region, improvement of local cattle by Dutch cattle and then by Holsteins
Kalmyk	KALM	native	1934	282.0	310.0	138.3	beef	South of Russia, Republic of Kalmykia; originates from cattle of nomadic people of the south steppe
Kholmogor	KHLM	native	1927	993.0	1388.5	222.9	dairy	North of the Europe part of Russia, Arkhangelsk region, islands in the upper reaches of the of the Northern Dvina river
Kostromsky	KSTR	locally developed	1944	276.0	158.0	10.9	dual purpose (milk/beef)	Central Russia, Kostroma region, crossbreeding local cattle with Brown Swiss cattle
Red Gorbatov	RGBT	native	1926	282.0	17.9	1.7	dairy	Central Russia, Nizhny Novgorod region; improvement by Tirol cattle (Tux-Zillertal)
Suksun	SKSN	locally developed	1943	23.3	13.7	2.3	dairy	Central Russia, Perm region; improvement of local cattle by Danish Red (Angeln cattle) and then by Red Holsteins
Yakut	YAKT	native	1929	2.3	0.9	0.6	primitive	Sakha (Yakutia) Republic; folk selection
Yaroslavl	YRSL	native	1925	951.0	413.3	50.5	dairy	Central Russia, Yaroslavl region
