# Supplementary material for: Whole-genome SNP analysis elucidates the genetic structure of Russian cattle and its relationship with Eurasian taurine breeds
Source: Genet Sel Evol. 2018 Jul 11;50:37. doi: 10.1186/s12711-018-0408-8 (PMC6042431; doi:10.1186/s12711-018-0408-8)
Supplement: Supplementary file 2 — Additional file 2: Table S2. Description of the reference samples included in the analyses. This table provides information about the reference samples used in this study including the sample size, country of origin, and the references where the genotypic data were previously published. [file 12711_2018_408_MOESM2_ESM.pdf]

Supplementary Materials

Table S2: Description of the reference samples included in the analyses

Region / Breed name	Abbreviations	Sample size	Country of origin	Reference
**Former USSR countries**				
Kazakh Whitehead	KAZAK	10	Kazakhstan	Present study
Auliatin	AULIA	9	Kazakhstan	Present study
Alataus	ALATA	6	Kazakhstan	Present study
Ukrainian Whitehead	WHUKR	11	Ukraine	Present study
Ukrainian Grey	GRUKR	4	Ukraine	Present study
**Great Britain**				
Red Poll	REDP	5	England	Decker J.I. et al., 2016
Angus	AN	15	Scotland	Decker J.I. et al., 2016
Scottish Highland	SCHL	8	Scotland	Decker J.I. et al., 2016
Hereford	HFD	15	Wales	Decker J.I. et al., 2016
Beef Shorthorn	SH	14	England	Decker J.I. et al., 2016
Jersey	JER	15	Jersey Island	Decker J.I. et al., 2016
**Northern Europe**				
Finnish Ayrshire	AYR	15	Finland	Decker J.I. et al., 2016
Norwegian Red	NRC	15	Norway	Decker J.I. et al., 2016
Holstein	HO	15	Netherlands	Decker J.I. et al., 2016
**Central Europe**				
Pinzgauer	PINZ	5	Austria	Decker J.I. et al., 2016
Gelbvieh	GEL	15	Germany	Decker J.I. et al., 2016
Simmental	SIM	15	Switzerland	Decker J.I. et al., 2016
Brown Swiss	BSW	12	USA	Decker J.I. et al., 2016
**Southern Europe**				
Limousin	LM	15	France	Decker J.I. et al., 2016
Charolais	CHA	15	France	Decker J.I. et al., 2016
Maine-Anjou	MAAN	5	France	Decker J.I. et al., 2016
Salers	SAL	15	France	Decker J.I. et al., 2016
Tarentaise	TARE	5	France	Decker J.I. et al., 2016
Piedmontese	PIED	15	Italy	Decker J.I. et al., 2016
Romagnola	RMG	15	Italy	Decker J.I. et al., 2016
Chianina	CHIA	9	Italy	Decker J.I. et al., 2016
Retinta	RET	4	Spain	Decker J.I. et al., 2016
Morucha	MOR	5	Spain	Decker J.I. et al., 2016

Region / Breed name	Abbreviations	Sample size	Country of origin	Reference
Pirenaica	PIR	5	Spain	Decker J.I. et al., 2016
**Turkey**				
Turkish Grey	TG	8	Turkey	Decker J.I. et al., 2016
Anatolian Black	AB	8	Turkey	Decker J.I. et al., 2016
South Anatolian Red	SAR	8	Turkey	Decker J.I. et al., 2016
East Anatolian Red	EAR	8	Turkey	Decker J.I. et al., 2016
**Eastern Asia**				
Hanwoo Korean cattle	HANW	8	Korea	Decker J.I. et al., 2016
Japanese Black	WAGY	12	Japan	Decker J.I. et al., 2016
Mongolian	MG	5	Mongolia	Decker J.I. et al., 2016
**China**				
Qinchuan	QC	4	China	Decker J.I. et al., 2016
Luxi	LX	5	China	Decker J.I. et al., 2016
Hainan	HN	4	China	Decker J.I. et al., 2016
**Australia**				
Murray Grey	MUGR	4	Australia	Decker J.I. et al., 2016
**Americas**				
Texas longhorn	TXLH	15	USA	Decker J.I. et al., 2016
Red Angus	ANR	15	USA	Decker J.I. et al., 2016
Beefmaster	BEFM	15	USA	Decker J.I. et al., 2016
Santa Gertrudis	SGT	15	USA	Decker J.I. et al., 2016
Brahman	BR	15	USA	Decker J.I. et al., 2016
Canchim	CANC	15	Brazil	Decker J.I. et al., 2016
Romosinuano	ROMO	8	Columbia	Decker J.I. et al., 2016
Corriente	CORR	5	Mexico	Decker J.I. et al., 2016
Nelore	NEL	15	Brazil	Decker J.I. et al., 2016
Senepol	SENP	15	Virgin Islands	Decker J.I. et al., 2016
**Africa**				
Africander	AFR	4	South Africa	Decker J.I. et al., 2016
N’Dama	NDAM	15	Ivory Coast	Decker J.I. et al., 2016
Sheko	SHK	15	Ethiopia	Decker J.I. et al., 2016
Boran	BOR	15	Ethiopia	Decker J.I. et al., 2016
Zebu Bororo	ZBO	15	Chad	Decker J.I. et al., 2016
Zebu Fulani	ZFU	15	Benin	Decker J.I. et al., 2016
Zebu from Madagascar	ZMA	15	Madagascar	Decker J.I. et al., 2016
**Southern Asia**				
Guzerat	GUZ	3	India	Decker J.I. et al., 2016
Gir	GIR	15	India	Decker J.I. et al., 2016

Region / Breed name	Abbreviations	Sample size	Country of origin	Reference
Ongole Grade	ONG	9	India	Decker J.I. et al., 2016
Hariana	HAR	10	India	Decker J.I. et al., 2016
Kankrej	KAN	10	India	Decker J.I. et al., 2016
Sahiwal	SAHW	15	Pakistan	Decker J.I. et al., 2016
Tharparkar	THA	12	Pakistan	Decker J.I. et al., 2016
Bhagnari	BAG	10	Pakistan	Decker J.I. et al., 2016
**Indonesia**				Decker J.I. et al., 2016
Bali	BALI	15	Indonesia	Decker J.I. et al., 2016
Aceh	ACE	12	Indonesia	Decker J.I. et al., 2016
Pesisir	PES	6	Indonesia	Decker J.I. et al., 2016
Madura	MAD	7	Indonesia	Decker J.I. et al., 2016
Brebes	BRE	9	Indonesia	Decker J.I. et al., 2016
