# Supplementary figures and images for: Whole-genome SNP analysis elucidates the genetic structure of Russian cattle and its relationship with Eurasian taurine breeds
Source: Genet Sel Evol. 2018 Jul 11;50:37. doi: 10.1186/s12711-018-0408-8 (PMC6042431; doi:10.1186/s12711-018-0408-8)

# Supplementary Materials

Additional file 3. Figure S1: CV-error according to K-number

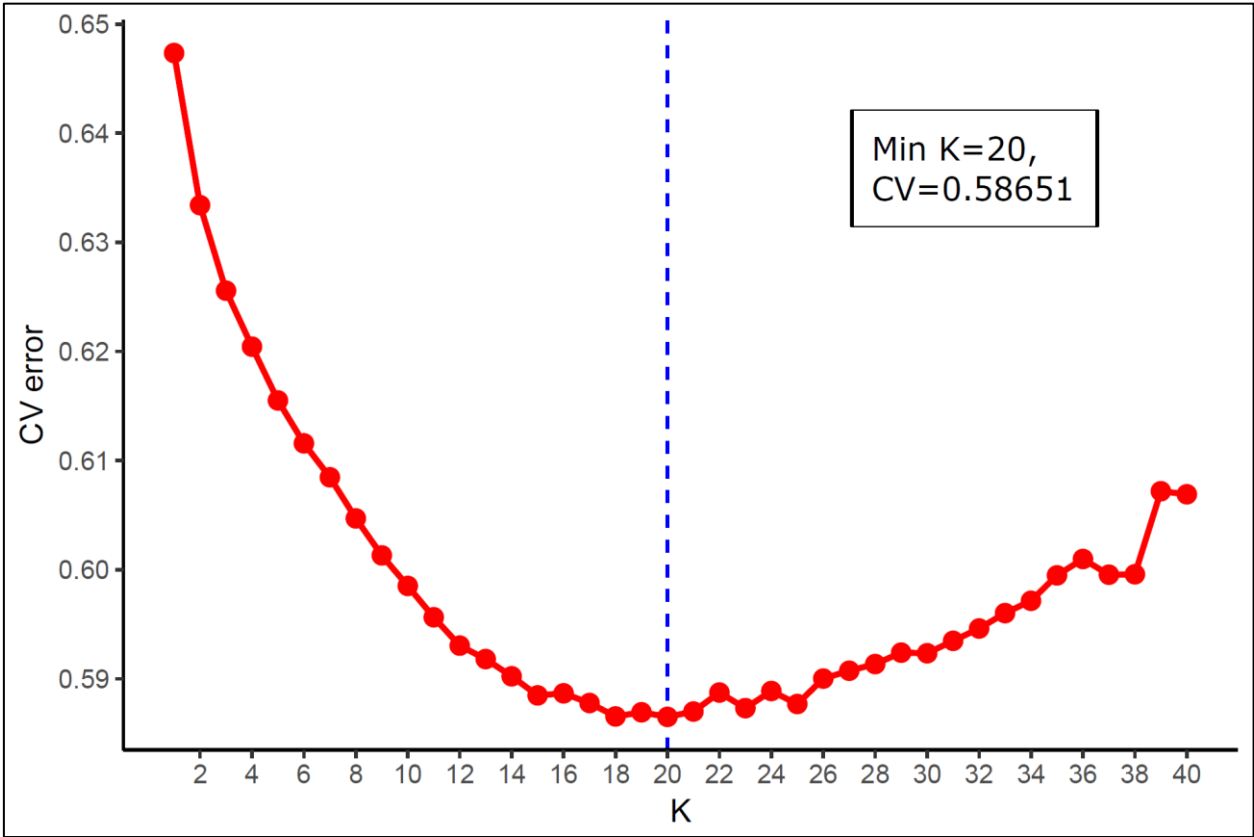

Supplement: Supplementary file 3 — Additional file 3: Figure S1. CV-error according to the K-number. This graph shows the CV-error for the number of clusters (K) from 2 to 40. [file 12711_2018_408_MOESM3_ESM.pdf]
