# Supplementary material for: Whole-genome SNP analysis elucidates the genetic structure of Russian cattle and its relationship with Eurasian taurine breeds
Source: Genet Sel Evol. 2018 Jul 11;50:37. doi: 10.1186/s12711-018-0408-8 (PMC6042431; doi:10.1186/s12711-018-0408-8)
Supplement: Supplementary file 4 — Additional file 4: Figure S2. Trend in slope changes of historical effective population size (Ne). This graph shows the trend in slope changes of the historical effective population size (Ne) for the period starting approximately 70 generations ago. For the full definition of breeds, see Table S1 [see Additional file 1: Table S1]. [file 12711_2018_408_MOESM4_ESM.pdf]

Supplementary Materials

Additional file 4. Figure S2: Historical effective population size (Ne) trend slope changes

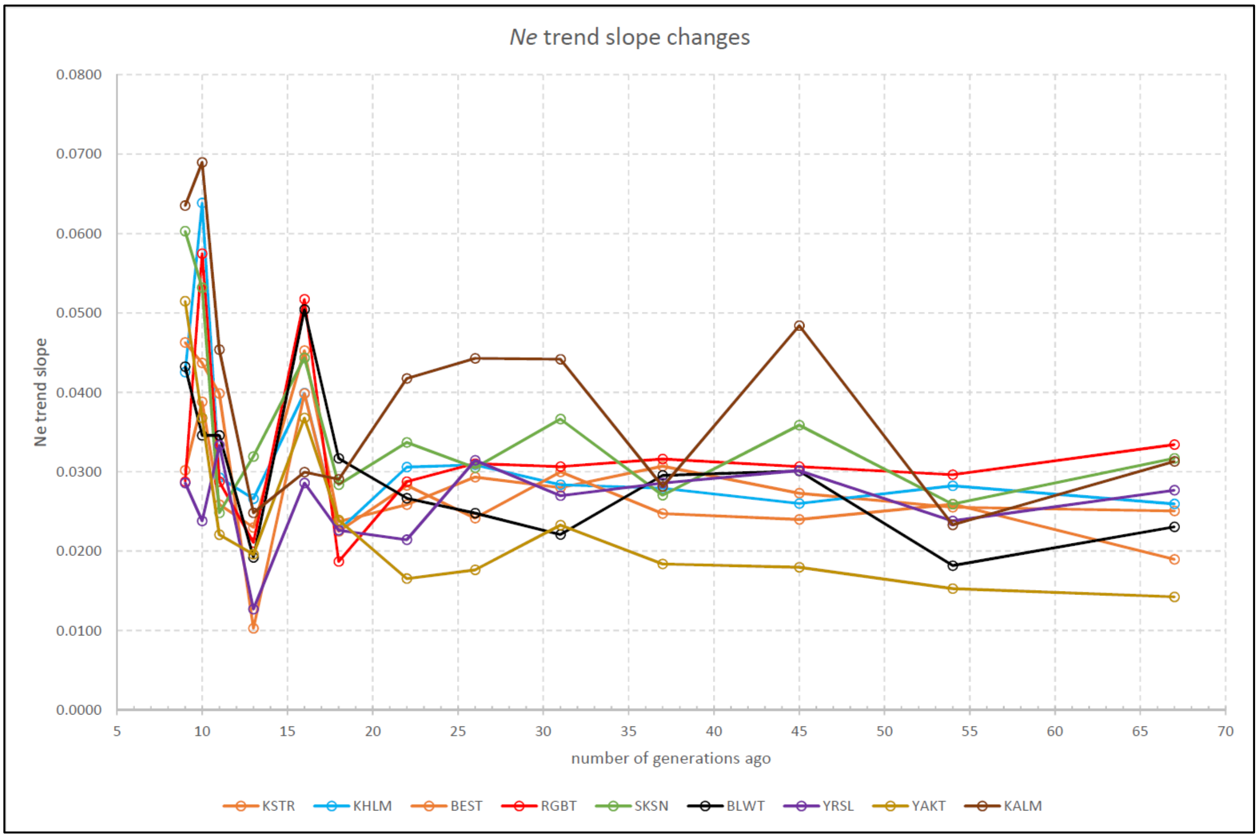

The results are shown for the period starting from approximately 70 generations ago. For full definitions of breeds, see Additional file 1, Table S1.
