# Supplementary material for: Whole-genome SNP analysis elucidates the genetic structure of Russian cattle and its relationship with Eurasian taurine breeds
Source: Genet Sel Evol. 2018 Jul 11;50:37. doi: 10.1186/s12711-018-0408-8 (PMC6042431; doi:10.1186/s12711-018-0408-8)
Supplement: Supplementary file 5 — Additional file 5: Table S3. Pairwise Wright fixation index (FST). This table provides the values of Wright’s fixation index (FST) at pairwise comparisons between the nine Russian cattle breeds and worldwide reference breeds. For the full definition of breeds, see Tables S1 and S2 [see Additional file 1: Table S1 and Additional file 2: Table S2]. [file 12711_2018_408_MOESM5_ESM.pdf]

Supplementary Materials

Additional file 5. Table S3: Pair-wise Wright fixation index (F_{ST})

	BEST	BLWT	KALM	KHLM	KSTR	RGBT	SKSN	YAKT	YRSL
BEST									
BLWT	0.0444								
KALM	0.0512	0.0638							
KHLM	0.0693	0.0699	0.0790						
KSTR	0.0738	0.0850	0.0722	0.1010					
RGBT	0.0744	0.0779	0.0810	0.0986	0.1029				
SKSN	0.0441	0.0360	0.0619	0.0708	0.0797	0.0594			
YAKT	0.1611	0.1771	0.1293	0.1858	0.1815	0.1901	0.1749		
YRSL	0.0680	0.0770	0.0701	0.0880	0.0946	0.0988	0.0743	0.1776	
KAZAK	0.0632	0.0702	0.0622	0.0910	0.0872	0.0929	0.0679	0.1842	0.0895
AULIA	0.0275	0.0243	0.0267	0.0548	0.0554	0.0596	0.0310	0.1479	0.0545
ALATA	0.0579	0.0670	0.0547	0.0898	0.0444	0.0909	0.0650	0.1809	0.0825
WHUKR	0.0490	0.0524	0.0476	0.0737	0.0706	0.0798	0.0543	0.1648	0.0714
GRUKR	0.0439	0.0535	0.0328	0.0773	0.0690	0.0807	0.0543	0.1611	0.0697
LM	0.0635	0.0750	0.0619	0.0930	0.0703	0.0939	0.0728	0.1768	0.0876
CHA	0.0643	0.0722	0.0658	0.0926	0.0815	0.0943	0.0693	0.1821	0.0913
SIM	0.0743	0.0866	0.0728	0.1046	0.0745	0.1048	0.0844	0.1861	0.0986
CHIA	0.0687	0.0777	0.0568	0.0980	0.0873	0.0989	0.0759	0.1735	0.0928
GEL	0.0721	0.0814	0.0717	0.1004	0.0819	0.1013	0.0800	0.1858	0.0958
MAAN	0.0803	0.0899	0.0942	0.1147	0.1162	0.1162	0.0868	0.2265	0.1179
PIED	0.0488	0.0583	0.0428	0.0759	0.0557	0.0769	0.0555	0.1569	0.0699
PINZ	0.0600	0.0700	0.0562	0.0922	0.0740	0.0915	0.0686	0.1836	0.0841
RMG	0.0927	0.1034	0.0770	0.1212	0.1051	0.1222	0.1022	0.1873	0.1150
SAL	0.0759	0.0869	0.0776	0.1050	0.0873	0.1059	0.0841	0.1913	0.1010
TARE	0.0690	0.0794	0.0678	0.1018	0.0750	0.1018	0.0784	0.1963	0.0941
HO	0.0554	0.0353	0.0788	0.0854	0.1002	0.0922	0.0359	0.1936	0.0908
AYR	0.0795	0.0816	0.0930	0.1049	0.1115	0.1084	0.0692	0.2084	0.1077
JER	0.1310	0.1392	0.1373	0.1556	0.1471	0.1579	0.1362	0.2469	0.1533
BSW	0.1034	0.1150	0.1036	0.1333	0.0522	0.1342	0.1084	0.2172	0.1257
NRC	0.0631	0.0598	0.0765	0.0881	0.0969	0.0929	0.0556	0.1942	0.0915
RET	0.1480	0.1574	0.1498	0.1795	0.1725	0.1813	0.1569	0.2746	0.1761
MOR	0.1403	0.1497	0.1374	0.1696	0.1616	0.1733	0.1492	0.2597	0.1647
PIR	0.0494	0.0572	0.0429	0.0788	0.0650	0.0813	0.0570	0.1716	0.0727
AN	0.0900	0.0956	0.1013	0.1151	0.1178	0.1165	0.0923	0.2153	0.1183
HFD	0.1082	0.1151	0.1156	0.1344	0.1324	0.1368	0.1124	0.2291	0.1338
REDP	0.0894	0.0956	0.1039	0.1210	0.125	0.1228	0.0943	0.2323	0.1212
SCHL	0.1129	0.1220	0.1216	0.1400	0.1388	0.1409	0.1177	0.2417	0.1370
SH	0.1099	0.1231	0.1356	0.1445	0.1549	0.1436	0.1169	0.2524	0.1520
TG	0.0706	0.0839	0.0407	0.1012	0.0894	0.1028	0.0818	0.1519	0.0914
AB	0.0809	0.0955	0.0445	0.1118	0.1016	0.1161	0.0935	0.1523	0.1008
SAR	0.0970	0.1094	0.0611	0.1271	0.1179	0.1301	0.1089	0.1712	0.1171
EAR	0.0819	0.0959	0.0461	0.1129	0.0999	0.1155	0.0944	0.1529	0.1018

HANW	0.0921	0.1073	0.0580	0.1210	0.1138	0.1248	0.1050	0.1424	0.1113
WAGY	0.1481	0.1631	0.1295	0.1772	0.1728	0.1810	0.1605	0.2105	0.1704
MG	0.0624	0.0759	0.0307	0.0944	0.0797	0.0963	0.0749	0.1362	0.0844
QC	0.0923	0.1075	0.0512	0.1277	0.117	0.1287	0.1062	0.1518	0.1147
HN	0.2811	0.2973	0.2550	0.3144	0.3138	0.3187	0.2933	0.3523	0.3076
LX	0.1612	0.1759	0.1198	0.1944	0.1881	0.1982	0.1738	0.2195	0.1838
GUZ	0.2603	0.2759	0.2295	0.2950	0.2918	0.2981	0.2722	0.3366	0.2873
GIR	0.3079	0.3292	0.2920	0.3385	0.3405	0.3438	0.3251	0.3665	0.3359
ONG	0.2742	0.2930	0.2513	0.3064	0.3058	0.3112	0.2893	0.3389	0.3010
HAR	0.3335	0.3551	0.3253	0.3637	0.3683	0.3695	0.3511	0.4000	0.3627
KAN	0.3014	0.3220	0.2840	0.3326	0.3344	0.3376	0.3170	0.3646	0.3293
SAHW	0.3076	0.3299	0.2924	0.3388	0.3410	0.3447	0.3257	0.3674	0.3366
THA	0.2904	0.3110	0.2704	0.3215	0.3221	0.3265	0.3067	0.3510	0.3175
BAG	0.2834	0.3023	0.2636	0.3145	0.3157	0.3199	0.2987	0.3482	0.3113
BALI	0.4690	0.5020	0.5157	0.5000	0.5176	0.5115	0.4971	0.5473	0.5104
ACE	0.3042	0.3250	0.2877	0.3356	0.3371	0.3411	0.3207	0.3660	0.3326
PES	0.2975	0.3159	0.2780	0.3298	0.3301	0.3349	0.3114	0.3676	0.3259
MAD	0.2839	0.3022	0.2638	0.3165	0.3162	0.3217	0.2988	0.3541	0.3116
BRE	0.2823	0.3017	0.2620	0.3145	0.3145	0.3201	0.2977	0.3485	0.3103
MUGR	0.0959	0.1002	0.1073	0.1260	0.1296	0.1283	0.0994	0.2392	0.1267
NDAM	0.1851	0.2044	0.1656	0.2134	0.2067	0.2198	0.2011	0.2598	0.2075
BOR	0.2145	0.2330	0.1856	0.2448	0.2415	0.2493	0.2288	0.2745	0.2386
SHK	0.1757	0.1936	0.1406	0.2043	0.1977	0.2086	0.1894	0.234	0.1971
AFR	0.1985	0.2136	0.1738	0.2298	0.2234	0.2338	0.2104	0.2823	0.2222
ZBO	0.1892	0.2072	0.1541	0.2186	0.2131	0.2232	0.2032	0.2463	0.2117
ZFU	0.2008	0.2196	0.1686	0.2302	0.2259	0.2358	0.2162	0.2584	0.2237
ZMA	0.2486	0.2699	0.2269	0.279	0.2765	0.2839	0.2646	0.3102	0.2741
CORR	0.0719	0.0804	0.0648	0.1002	0.0919	0.1036	0.0796	0.1927	0.0955
TXLH	0.0722	0.0820	0.0638	0.0977	0.0897	0.1014	0.0792	0.1774	0.0943
SENP	0.0607	0.0604	0.0628	0.0886	0.0899	0.0913	0.0592	0.175	0.0881
ROMO	0.0961	0.1061	0.0915	0.1248	0.1183	0.1256	0.1041	0.2118	0.1218
ANR	0.0939	0.1005	0.1063	0.1210	0.1233	0.1227	0.098	0.2209	0.1235
BR	0.2694	0.2886	0.2466	0.2996	0.3001	0.3046	0.2845	0.3297	0.2958
NEL	0.3087	0.3303	0.2930	0.3398	0.3418	0.3451	0.3259	0.3679	0.3368
BEFM	0.0892	0.1006	0.0773	0.1186	0.1162	0.1202	0.0966	0.1874	0.1155
SGT	0.0872	0.0985	0.0802	0.1189	0.1195	0.1188	0.0944	0.1902	0.1182
CANC	0.0729	0.0859	0.0550	0.1032	0.0916	0.1048	0.0820	0.1635	0.0978

Note: For full definitions of breeds, see Additional file 1, Table S1 and Additional file 2, Table S2.
